# Utility and usability evaluation of an information diary tool to measure health information access and exposure among patients with high cardiovascular risk

**DOI:** 10.3389/fpubh.2023.1132397

**Published:** 2023-05-09

**Authors:** Hooi Min Lim, Chirk Jenn Ng, Adina Abdullah, Jason Dalmazzo, Woei Xian Lim, Kah Hang Lee, Adam G. Dunn

**Affiliations:** ^1^Department of Primary Care Medicine, Faculty of Medicine, University of Malaya, Kuala Lumpur, Malaysia; ^2^Department of Research, SingHealth Polyclinics, Singapore, Singapore; ^3^Duke-NUS Medical School, Singapore, Singapore; ^4^Biomedical Informatics and Digital Health, School of Medical Sciences, Faculty of Medicine and Health, The University of Sydney, Sydney, NSW, Australia

**Keywords:** consumer health information, access to information, information-seeking behavior, user-centered design, health communication

## Abstract

**Background:**

Online health misinformation about statins potentially affects health decision-making on statin use and adherence. We developed an information diary platform (IDP) to measure topic-specific health information exposure where participants record what information they encounter. We evaluated the utility and usability of the smartphone diary from the participants' perspective.

**Methods:**

We used a mixed-method design to evaluate how participants used the smartphone diary tool and their perspectives on usability. Participants were high cardiovascular-risk patients recruited from a primary care clinic and used the tool for a week. We measured usability with the System Usability Scale (SUS) questionnaire and interviewed participants to explore utility and usability issues.

**Results:**

The information diary was available in three languages and tested with 24 participants. The mean SUS score was 69.8 ± 12.9. Five themes related to utility were: IDP functions as a health information diary; supporting discussion of health information with doctors; wanting a feedback function about credible information; increasing awareness of the need to appraise information; and wanting to compare levels of trust with other participants or experts. Four themes related to usability were: ease of learning and use; confusion about selecting the category of information source; capturing offline information by uploading photos; and recording their level of trust.

**Conclusion:**

We found that the smartphone diary can be used as a research instrument to record relevant examples of information exposure. It potentially modifies how people seek and appraise topic-specific health information.

## 1. Introduction

The increase in the number of sources and conduits for health information means that there are now many more ways to access health information, both credible and evidence-based, as well as low-quality or misleading (1). The health information people are exposed to has a complex influence on their self-care and decision-making. Good health information increases patients' understanding of their diseases, participation in shared decision-making and adherence to treatment (2). People who seek health information have a higher intention to adopt healthy lifestyles (3). However, exposure to low-quality health information can negatively affect health behaviors (4). Frequent online health information-seeking has been shown to associate with lower medication adherence (5). During the COVID-19 pandemic, exposure to online misinformation is correlated to vaccination hesitancy and refusal (6). The World Health Organization (WHO) recently issued a call to action in the area of infodemic management, including recommendations related to finding new ways to use digital technologies to promote access to good information and to measure the burden of the infodemic on behaviors (7).

People can obtain health information either actively or passively (8, 9). Active health information-seeking is a goal-orientated activity where people seek specific health-related information, and includes searching online or a consultation with a health professional (10). Passive health information exposure occurs when people see, read, or hear health-related information while doing other activities, including via traditional media advertising or while browsing social media online (11, 12). Health behavior research has been attempting to measure health information-seeking and exposure, and relate it to health behaviors. However, the relationship remains variable; for example, a recent systematic review published by the authors found no association between health-information-seeking and medication adherence, and raised concern regarding the validity of the research instruments in capturing the health information-seeking behavior (13).

Traditionally, information access and exposure can be measured by asking participants to recall sources as part of surveys conducted at a single point (2, 14). An alternative tool is a media use diary, where participants record what they see as they encounter it, which can enable prospective studies and studies that better examine information exposure over time. Media use diaries, while less likely to lead to recall bias, require efforts from the participants in writing and logging their information diaries (15). More recently, there has been a range of studies that use data from social media at scale to estimate information exposure. However, these studies do not recruit participants, and hence remain disconnected from their health behaviors (16, 17). For studies that recruit participants, there is a lack of standardized approaches and inconsistency in how information access and exposure are measured (2, 13). The wide variations in how research captured health information-seeking behavior make data synthesis and comparison across studies challenging.

Statin use has been extensively debated in mainstream media and social media since 2013 when an article was published stating that statin side effects are much higher than reported in clinical trials (18). In the online platforms, statins received a predominantly negative portrayal that was disproportionately biased toward adverse effects of treatment, questioning the reliability of research evidence with potential pharmaceutical industry influence (19). As a result, several cardiovascular health studies reported that statin-related health misinformation is associated with statin non-adherence (20, 21). Online health misinformation about statins potentially affects health decision-making on statin use for cardiovascular disease prevention (22, 23).

To address these challenges, we developed the Information Diary Platform (IDP) which is a web-based application that includes a smartphone-based diary tool and a researcher dashboard. The smartphone-based diary tool was designed to be simple to use and allowed study participants to record their access and exposure to topic-specific health information. Research participants capture the information sources and rate how much they trust each source as they enter a new record. The research dashboard allows researchers to tailor the diary tool according to the health topic and sources of information they wish to study. In this study, using statin adherence as an example, we evaluated the utility and usability of the IDP diary tool for recording topic-specific health information access and exposure among patients with high cardiovascular risks.

## 2. Methods

### 2.1. Information diary platform

**Figure 1:**
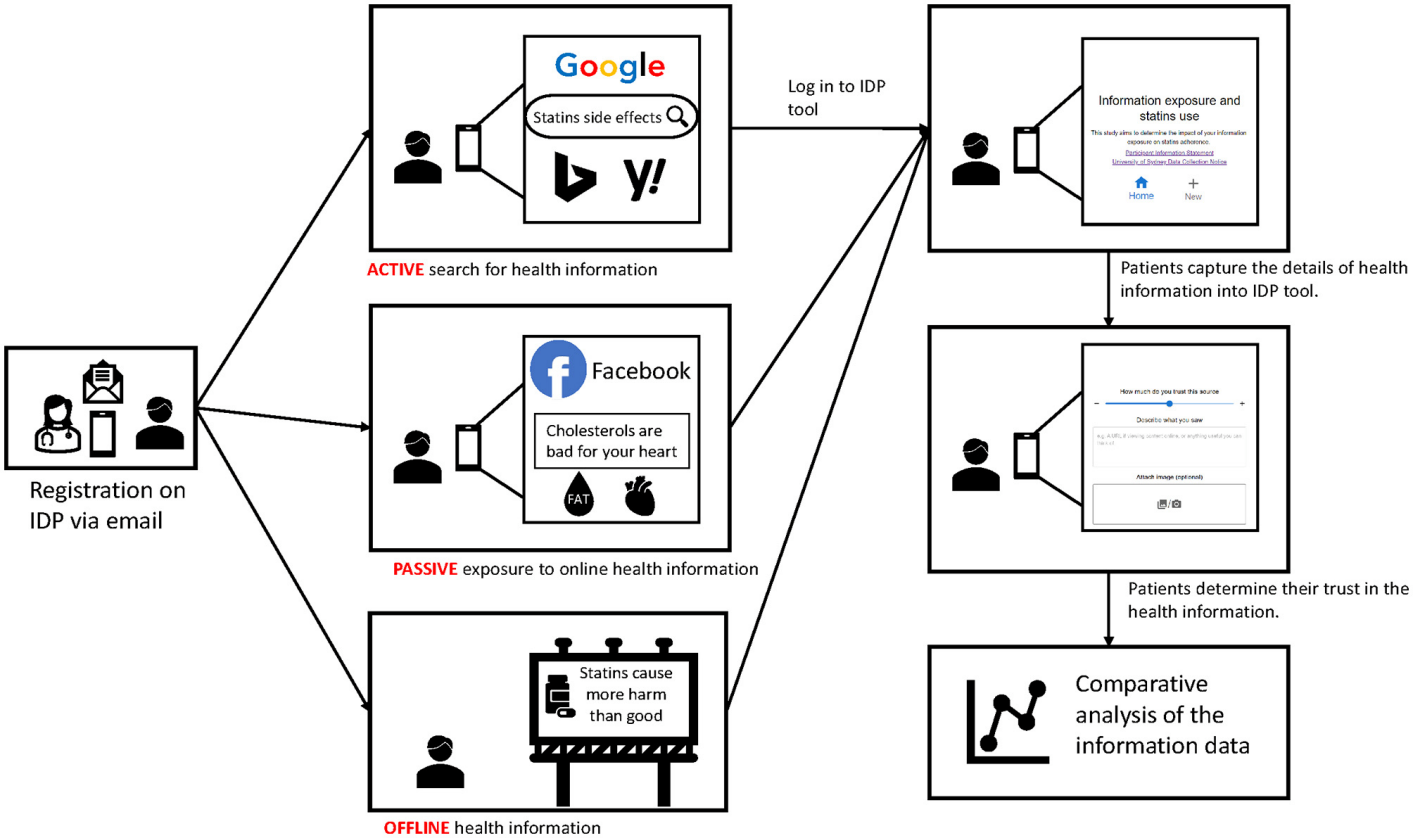
This storyboard illustrates the process of capturing health information using an information diary platform (IDP) from a user perspective.

The IDP is a web-based research support platform used to design and administer studies that measure health information access and exposure. The diary tool can be used on smartphones or any other device with a web browser to capture health information access and exposure. When participants encounter a piece of health information, they log in to the diary tool and record the health information as a new entry (Figure 1). The IDP includes a researcher dashboard to support the delivery of the smartphone diary tool. The researcher dashboard is a web-based platform with templates for researchers to customize the smartphone diary tool to match their topic and study design. Study investigators can enter their study details into the researcher dashboard and access the data when authenticated on the platform.

**Figure 2:**
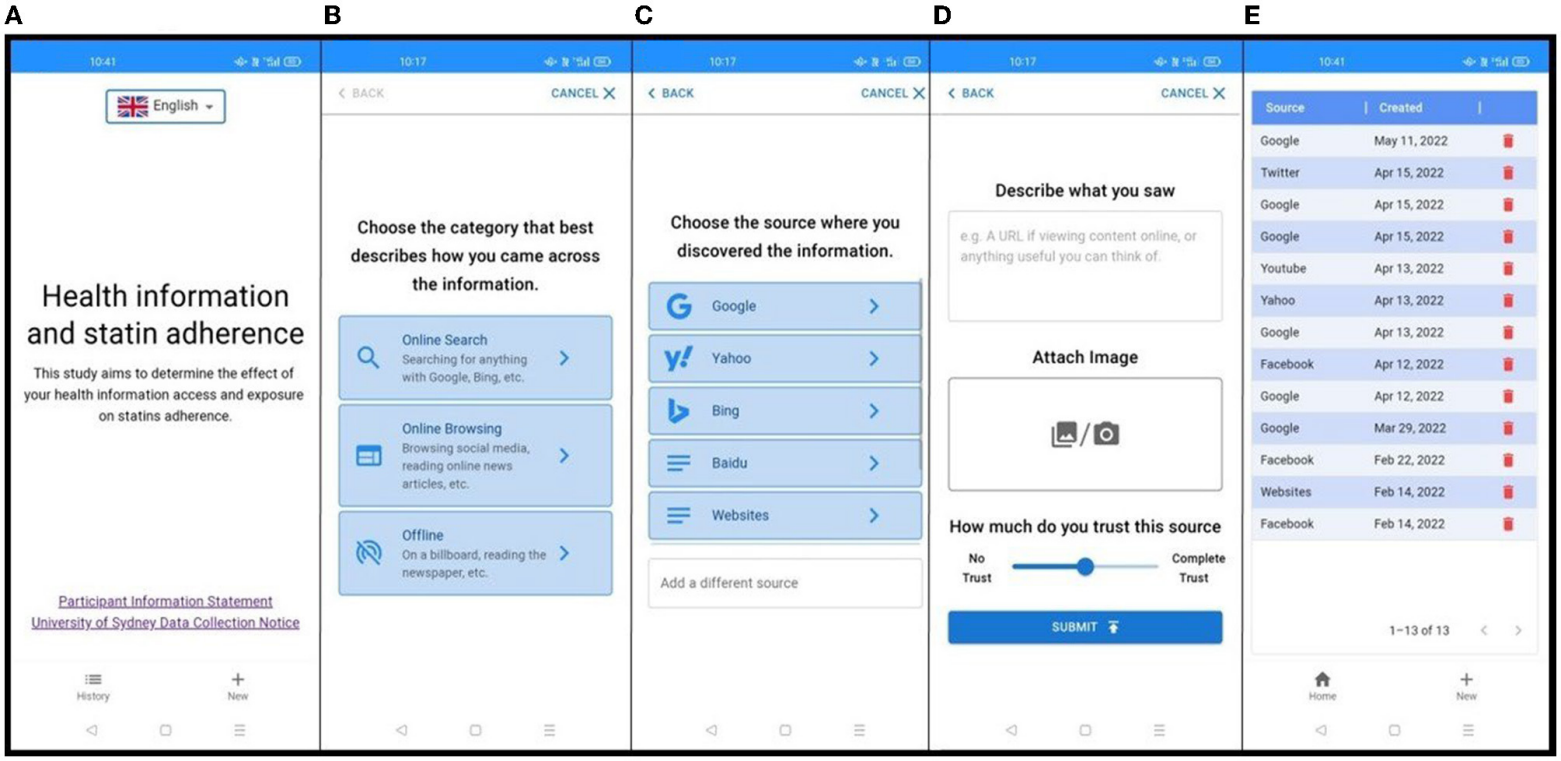
Screenshots of the information diary tool including (from left to right): **(A)** home page of the diary tool; **(B)** choosing the information category; **(C)** selecting the information source; **(D)** determining the trust level of information; **(E)** history page.

The IDP captured category and source of health information, as well as the participant's perception of how much they trust the information (Figure 2). Firstly, the participant was given three categories of health information sources to choose: (a) “Online search”: when they were actively searching for topic-specific health information via a search engine; (b) “Online browsing”: when they encountered relevant health information while browsing social media, online news, and other webpages; and (c) “Offline”: when they encountered information offline, such as printed advertisements or in conversations with health professionals. Secondly, the participant selected a sub-category, which included some predefined examples of sources such as YouTube, Facebook, newspapers and friends. Participants could also add a new source under the sub-category. The participant was encouraged to write a short description of the health information or copy a URL of information from the Internet. For offline information, participants could attach an image of the health information. Finally, the participant rated their trust level for each health information they recorded. Participants were able to access a history page showing the details of their previous entries.

### 2.2. Study design

We used a convergent mixed-methods study design to evaluate the utility and usability of the diary tool from the perspective of the participants enrolled in this study. A mixed-methods study design supports a comprehensive view of the usability challenges in different domains, including an in-depth understanding of usability problems that can be used to revise and improve the diary tool. Our quantitative component was an evaluation of the usability of the information diary tool using a System Usability Scale (SUS) questionnaire and measured how users interacted with the information diary tool via metadata stored by the IDP during the pilot study. Our qualitative component was a set of interviews using a think-aloud method. Both quantitative and qualitative data were collected at the same time, and data were analyzed and integrated to extend the breadth of our understanding of the usability issues.

### 2.3. Study setting and study population

This study was approved by the University of Malaya Medical Center Medical Research Ethics Committee (MECID No: 2021324-9981). Written informed consent was obtained from all individual participants included in the study. We conducted this study at an urban primary care clinic in the University Malaya Medical Center (UMMC) in Kuala Lumpur, Malaysia. Kuala Lumpur is the capital city in Malaysia and the main languages used are Bahasa Malaysia, English, and Mandarin.

We recruited patients with high cardiovascular risk attending the primary care clinic during the recruitment period. The inclusion criteria were: (a) aged ≥ 18 years; (b) patients who had high cardiovascular risk where statin use was indicated as per guideline (10), including those with pre-existing cardiovascular disease (CVD), diabetes mellitus, chronic kidney disease ≥ stage 3, and Framingham General CVD risk score ≥ 20%; and (c) patients who owned and used a smartphone. The exclusion criteria were: (a) patients who were unable to read English, Malay, or Mandarin; and (b) patients who were too ill or cognitively impaired to participate.

We purposively sampled participants from different age groups, gender, ethnicity, language, and educational levels to achieve maximum variation. The sample size of a usability study is typically small (around 5–10 participants) (24); as the information diary tool was made available in three languages, we aimed to recruit 5–10 participants per language. We stopped recruiting participants when data saturation was reached after 24 interviews where no new themes emerged from data analysis and field notes.

### 2.4. Data collection

Researchers briefed the participants about the study objectives and method using a participant information sheet. Researchers encouraged participants to ask questions and informed the participants that their participation was voluntary and that they could leave the study at any time. Once participants agreed to participate, they were asked to sign a consent form and complete a form that captured their demographic characteristics.

A video and infographic user guides were developed to teach the participants how to use the information diary tool with guidance from research assistants. Participants were asked to capture any relevant health information that they encountered for a week. At the end of the week, participants were invited to the clinic for a face-to-face interview. If participants were unable to attend the clinic physically, they were interviewed over video conferencing.

We used the System Usability Scale (SUS) questionnaire to assess the usability of the diary tool from the user perspective (25, 26). Before the start of the interview, participants were asked to answer the SUS questionnaire. Participants were then asked in an interview about their overall opinions of the tool and detailed questions about its functionality and ease of use. We developed a semi-structured interview guide (Appendix 1) based on Nielsen's model of usability (27). For the think-aloud method, we asked the participants to show us how they used the diary tool to capture information and think out loud about how they performed the tasks. We asked the participants to comment on how they captured examples of relevant information and discussed any problems they encountered. We also asked the participants for suggestions on how to improve the usability of the diary tool. The interviews were conducted by researchers (HML and WXL) trained in qualitative research and digital health research. All interviews were audio-recorded.

### 2.5. Evaluation measures and data analysis

The SUS questionnaire was used to assess usability after a week of testing by the participants. SUS (10 items rated on a 5-point Likert scale) was selected as it has been validated and widely used in digital health applications (25, 26). We reported the SUS results as a total score using mean and standard deviation, as well as with descriptive data for each item. An SUS score of above 68/100 is considered as good usability (25). To evaluate how users recorded entries using the diary tool, we extracted their submission data from the IDP researcher dashboard, which contains data on the number of entries for each participant, categories of information source, and levels of trust for each record.

After each interview, researchers listed the issues and comments on the field notes. All audio recordings were transcribed verbatim and checked independently by a researcher (HML). We used a thematic approach to analyze the data (28). For the first two transcripts, two researchers (HML, WXL) familiarized themselves with the data by reading and re-reading the transcripts and field notes and generating initial codes independently. Both researchers met to discuss and compare the codes and coding frame, together with other researchers (AGD and CJN). Any differences between the codes were solved through consensus. One researcher (HML) then coded the remaining transcripts. The research team met regularly to discuss new codes and emerging themes. The qualitative data analysis was performed using NVivo version 10.

## 3. Results

### 3.1. Study participants and IDP usage

**Figure 3:**
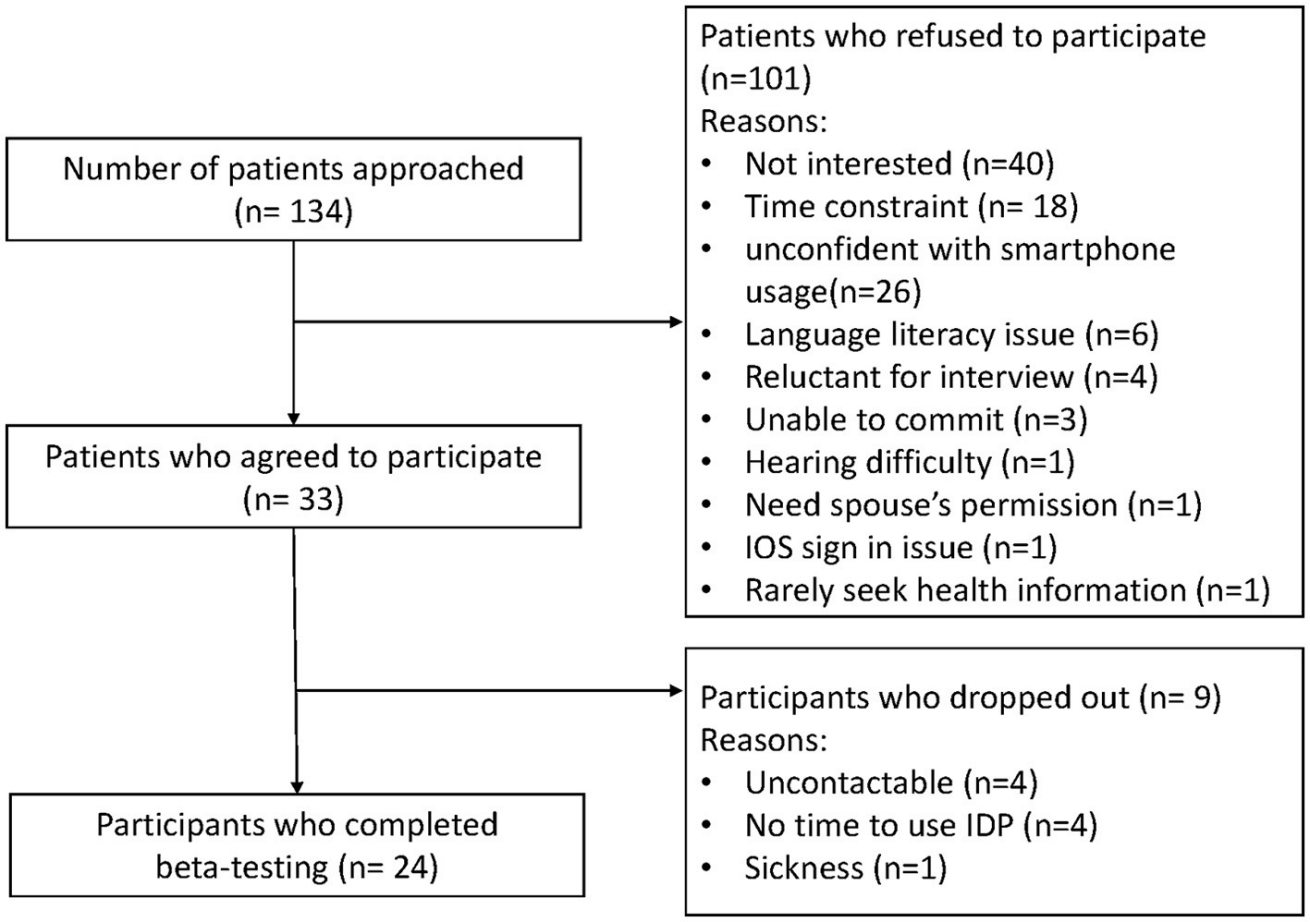
From 134 patients approached to participate in the study, 24 completed the evaluation.

A total of 134 eligible patients were approached and invited to participate in this study (Figure 3). Thirty-three participants agreed to participate. The main reasons why eligible patients chose not to participate were lack of interest in participating in a research, time constraints, and lack of confidence with using a smartphone. Of the 33 participants who agreed, 24 completed a week of testing and participated in the interviews. Most participants were over 50 years old and had an educational level above secondary school (Table 1).

**Table 1:** Characteristics of participants (*n* = 24).

**Characteristics**	***N* (%)**
**Age (years)**	
30–49	8 (33.3)
50–69	15 (62.5)
70–79	1 (4.2)
**Gender**	
Male	9 (37.5)
Female	15 (62.5)
**Ethnicity**	
Malay	7 (29.2)
Chinese	11 (45.8)
Indian	4 (16.7)
Others	2 (8.3)
**Language**	
English	9 (37.5)
Malay	9 (37.5)
Chinese	6 (25)
**Educational level**	
Lower secondary school	1 (4.2)
Upper secondary school	4 (16.7)
Pre-Uni/A-level	2 (8.3)
Diploma/Degree	11 (45.8)
Master/PhD	6 (25)

### 3.2. Utility of IDP

Five themes emerged from the qualitative analysis related to the utility of the tool (Table 2, includes participant quotes). These are: (1) IDP functions as a health information diary; (2) use of the history function in supporting discussion of health information with doctors; (3) participants wanting a feedback function about credible information; (4) the diary tool increasing awareness of the need to appraise information; and (5) participants wanting to compare levels of trust with other participants or experts.

**Table 2:** Themes and subthemes related to the functionality of the information diary tool.

**Theme**	**Subtheme**	**Sample quotes**
IDP functions as a health information diary	To retrieve and re-read health information	*Archive, you can archive it. It's good. For me, I think history is good… I have read something and I want to go back to it, but I can't remember where. So, if there is this thing pertaining to medical, and I have saved it there, I can go back to the history, and I can retrieve and look at it, and find where did I read it. (P7, 58-year-old)* *It is time-wasting if you need to search information online. If I can save it in this app, I don't have to waste time searching for the information. For example, I can search for health information online while waiting for my friends, and then I can read back the information during my break time. (P22, 58-year-old)*
	To compare recent and previous information	*Sometimes I want to double confirm or compare with other information (P16, 32-year-old)*
	Suggest categorizing information according to diseases or topics	*Yeah, for me, the source is not important, whether it's Google or YouTube, it's not important. The nature of the data, the description, whether it's your musculoskeletal system, or digestive system, which part…So if I categorize it according to my system, then easy for me to go in and use it. (P1, 61-year-old)* *I prefer the app to be categorized into topics, maybe as weight loss, cholesterol, and anemia. If there are such topics, it's easier for me to refer to. (P15, 41-year-old)*
	Suggest retrieving information using keywords	*I would love to go back to what I read something but I just can't remember. So maybe there is an area where I said I've read something about lingzhi so I just type lingzhi and the thing will come out where all the info that I have stored and I can just go in. (P7, 58-year-old)*
Supporting discussion of health information with doctors	Show and discuss with doctors about health information using IDP	*I can show the doctor, compare medical information from other countries, consult about exercise. Also, I can show and ask the doctor about medical treatment options and their credibility. If I use this app, I can show the doctor directly. If I still need to look for it online, the doctor has no time to wait. (P22, 58-year-old)* *When I see my doctor, I can show him where I get my info from… I can click back ‘history', then show him that I get from here, I get from Google. (P12,64-year-old)*
	For healthcare providers to understand patients' health information-seeking	*To me, it is very useful. As I said, useful for you all (healthcare providers), actually to learn about people, how we all come across this kind of message, and how we can forward it to somebody…So, I think if it is useful to y'all. (P6, 59-year-old)*
Feedback function about credible information	Suggest providing credible health information	*Maybe it would be good if we could be given suggestions on other articles, linked to that, that we could read up on maybe to get more knowledge or if you think that the article. (P5, 56-year-old)* *Okay, maybe there's a feedback. The IDP would tell me, Okay, when you have this health information submitted, and probably there'll be another health information which IDP may recommend us to look at it…And different websites give you different information. (P4, 67-year-old)*
	Suggest healthcare professionals verify the credibility of information	*It's good to send you information like this. And you as a medical practitioner, maybe you all can verify which is true, which is not so we need to have some feedback from that. So that will be great if there is a feedback. (P6, 59-year-old)* *If my doctor thinks this website can be trusted, maybe I will trust it as well… Maybe showing the results, five out of ten medical professionals who saw this website also trusted it. (P24, 34-year-old)*
Increasing the awareness of the need to appraise information	IDP reminds participants to appraise health information	*Before this, I don't really care. I just read and I just trust everything, but when I use IDP because it is gonna differentiate the source of information. So when you read, it makes you think, to what extent should I trust this information. Compared to before, I just trust everything and I don't really check where this is coming from, who is writing it. I did check it now. (P18, 31-year-old)*
	Suggest providing guidance on how to determine the trustworthiness of information	*Yes, I think if in the app it would give extra information. They can provide in the app, like what kind of information, from what source should be trusted 90% or 100% or which one that is, you should not really trust that much. You know, so that at least we have like a guideline on how much should we trust the information. (P18, 31-year-old)*
Views about comparing their results with others	Compare their information diary with others	*We might not be so savvy on this. Some people are really good. They know where to search… and it's stored there and you do have a comparison. Then I can see how come he/she can get this interesting (information) that I will go in and look at the info. (P8, 47-year-old)* *If I was interested in a certain topic, I see what other people are reading then I might also go and read that information. (P9, 56-year-old)*
	Compare the level of trust	*It will help, let say, if I know this information, other people also trust, maybe it is quite trustable also. (P24, 34-year-old)*

#### 3.2.1. The IDP functions as a health information diary

In terms of its function as a health information diary, participants liked the idea of a specific application they can use to record and retrieve the health information that they have encountered. Participants expressed that they often forget the source of past information they have encountered, especially from social media and the diary tool helps them remember. Participants felt the tool is useful to compare information sources. Participants suggested an additional function to let them categorize or filter their “History” according to diseases, body systems, or topics rather than by source. Participants also suggested a search function or the ability to label entries so they could find them more easily later.

#### 3.2.2. Supporting discussion of health information with doctors

In terms of its use to support discussion with doctors, participants stated that they could bring the health information recorded in the diary tool to show and discuss with their doctors during clinic consultations. They were keen to consult their doctors about the information that they have encountered, especially where they were unsure about its credibility. They stated that the diary tool would save time during consultations, and that it would be useful for healthcare providers to be aware of the types of information they encounter.

#### 3.2.3. Feedback function about credible information

In terms of feedback about credible information, participants were interested in receiving feedback from the diary tool about the credibility of what they see, beyond just recording their own perceptions of trust. They wished that the diary tool would help assess the information they have encountered or provide credible alternative health information. Participants hoped to get feedback from healthcare professionals to verify the credibility of the information they accessed.

#### 3.2.4. Increasing the awareness of the need to appraise information

While recording the health information that they encountered, some participants revealed that they became more conscious of the need to critically appraise the credibility of health information when they entered their trust level into the tool. Participants wished to have more guidance on how to assess the credibility of information sources they encounter.

#### 3.2.5. Views about comparing their results with others

In terms of the views about the value of comparative analytics, participants liked the idea of having a report comparing their information diary with others. Most participants perceived that the information sources they accessed were limited, and they would like to know other information sources on a similar topic. A participant expressed that a comparison of the trust levels of others would help them to reflect on their own trust.

### 3.3. Usability of IDP

#### 3.3.1. System usability scale

**Figure 4:**
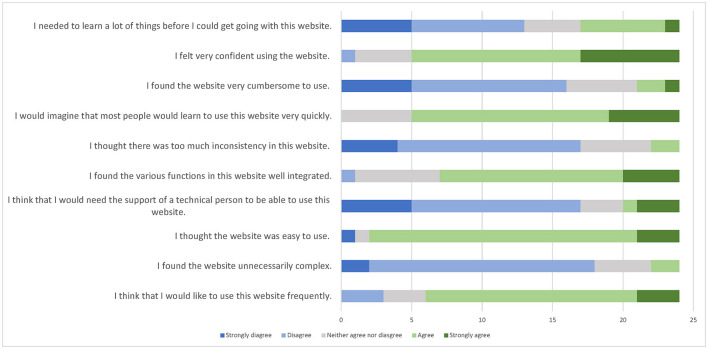
Results of the system usability scale (SUS) items for 24 participants.

All 24 participants completed the SUS questionnaire after testing the diary tool for a week. The mean score was 69.8, which indicated good usability (Figure 4). Of the 24 participants, 22 agreed that the tool was easy to use, 19 were confident using it, and 18 would use the tool frequently.

#### 3.3.2. Summary of IDP submissions

There was a total of 452 submissions of health information by the participants (Appendix 2). One participant did not submit any information because he did not understand the function of the diary tool. He was unclear about the function and instructions to use the diary tool. One participant had recorded 230 items because he often encountered health information when surfing through social media. The “online browsing” category had the most submissions (306 submissions, 67.7%), followed by the “online search” category (107 submissions, 23.7%) and then the “offline” category (39 submissions, 8.6%).

Four themes emerged from the interviews related to usability (Table 3, includes participant quotes). These were: (1) ease of learning and using the diary tool; (2) confusion about selecting the category of information source; (3) capturing offline information by uploading photos; and (4) recording their level of trust.

**Table 3:** Themes and subthemes related to the usability of the information diary tool.

**Theme**	**Subtheme**	***Sample quotes***
Ease of learning and using the diary tool	Easy to submit information without assistance	*For me, the process was actually relatively easy. (Name of assistant) explained it very well that day, and he also demonstrated how to use it. So I just went home and tested whether I can insert the words, insert the picture. On a scale of one to five with five being the easiest, I think I could probably say maybe 4.5. (P24, 34-year-old)*
	Older patients and those with lower IT skills were able to use IDP	*It was very easy. He (research assistant) showed me how to use it. I was a little bit apprehensive because I'm going to be 59 this year and. you know, not IT friendly people, and I was worried that whether I could do it…but surprisingly it was breeze. So, it was easy for me it was not a problem at all. (P3, 58-year-old)*
	A step-by-step visual guide was helpful	…*I can't remember what I did yesterday. I tried to recall what you (researcher) said…there's something that I missed but when you send me that video, I find it very good…So I go back and I go through it then I understand more… because sometimes we just tend to forget. (P7, 58-year-old)*
	Suggest a written material to explain IDP	*You give them a pamphlet to read before recruitment. Then, when you come back and talk to them again, they can understand better about this research and the structure of this app. (P14, 67-year-old)*
Confusion about selecting the category of information source	Confusion between “online search” and “online browsing”	*I just opened my Google Chrome. You see a lot of articles at the bottom will come out like which I may be interested in. So if I want to submit it to the IDP, then I'm not sure what category goes under. It's not offline. But on the other hand, I'm not searching actually. It's just there… so I just put it on what category where I could find Google. I don't know the correct category. (P9, 56-year-old)* …*I don't know which subcategory, I think I have searched it under Google…and then when I stored, I went and clicked on YouTube. (P1, 61-year-old)*
	Select the main category (online search or online browsing) based on the sub-category (name of sources)	*I have no idea. I just clicked inside, I look at the subcategory then I know what it is. But if I didn't click inside, I don't know which one it is. Yeah, for example, the information from Facebook, I don't know it is from the first category (online search) or the second category (online browsing). So once actually inside then I know. (P20, 42-year-old)*
	Suggest changing the wordings to active search and passive exposure	*There is a little overlapping. Because the first one says “online search”. Then the second one says “online browsing”. Searching and browsing, that is overlapping… Maybe you should be more specific. Online search—maybe you can put something like own initiative. Online browsing—would be like sent to you by someone. Meaning to say not that you take your own initiative, but someone sent you the health articles that you find relevant and useful. (P12, 64-year-old)*
Capturing offline information by uploading photos	Longer time to upload photos	*Sometimes I use my own camera, I save it to my gallery and then I just upload or sometimes I just do it directly from the app, but both ways are very slow. (P9, 56-year-old)*
	Unable to use the camera function embedded in IDP	*If I'm actually trying to take a photo using the app, that is where the app crashes, it closes. So I did not manage to take the photo and save it. (P24, 34-year-old)*
	Inconvenient to capture photos of offline information	*Okay, let's say when I am on my way to the pharmacy, a promoter comes in all these things, and I can't be taking photos. (P7, 58-year-old)*
Recording their level of trust	Unsure about their trust level of the information	*Yes, I don't know how much (trust) to put, so is usually about the middle. Because I am not familiar with medicine, I can't say I don't believe it and I can't believe it 100%. (P22,58-year-old)* *Is difficult to decide the level of trust. (P19, 74-year-old)*
	Determine trust level based on logical thinking, author and sources of information	*Our thinking is of an ordinary layman, if it's logical, we give a higher score. If there is no logic, we will put a lower score. (P19, 74-year-old)* *Whatsapp one, then I won't put a very high percentage, somewhere in the middle, cause those things cannot always be trusted. But Google, I generally trust maybe at about 75%. But definitely from the newspapers I will trust above 90%. (P12, 64-year-old)* *I almost trust. It depends on who writes the article, the topic? If he's a doctor, of course, they know about it, so I might trust more than 50 or 60%. If the normal person the layman it just maybe 30%-50%. (P8, 47-year-old)*
	Problems dragging the sliding scale of trust	*I think it is better to put a number. Because sometimes when I want to press, it will get stuck. Better out as numbers so that people can press directly. (P7,58-year-old)* *I can see that it would be easier if there are separate icons for like 10, 20, 30, 40, 50, 60%. Yeah, maybe they'll make it easier for others. (P24,34-year-old)*

#### 3.3.3. Ease of learning and using the diary tool

The participants agreed it was easy to learn and use the diary tool. They performed the tasks of submitting their information diary independently without assistance, including those participants who were older and less familiar with mobile technology. Participants found that the short video and visual guide explaining the step-by-step guide were helpful. One participant had difficulty grasping the concept and objectives of the diary tool and suggested a written pamphlet to explain the functions.

#### 3.3.4. Confusion about selecting the category of information source

Participants reported being confused about the selection of categories and subcategories. For example, uncertainty occurred when participants were actively searching for health information from Google, but the Google search results eventually led them to YouTube or other social media applications. Sometimes, participants were being “pushed” with information from Google without actively searching for it, and they were confused whether to choose “online search” or “online browsing” because “Google” is under the category of “online search”. Due to the confusion of the main categories, some participants chose the category based on the sub-category listed. Some participants complained about having too many subcategories listed. Participants suggested changing the main categories to be more aligned with active searching or passive exposure. One of the participants did not upload their information diary from WeChat because the option was not listed, and they were unsure how to add a new source.

#### 3.3.5. Capturing offline information by uploading photos

When participants encountered offline information such as newspapers, billboards, or paper-based written materials, they were encouraged to write a short description of the information or attach a photo showing a snippet of information. Participants faced challenges when attempting to upload photos using the tool because uploading could be slow with slow internet speeds. In addition, some participants could not use the camera function embedded in the diary tool, requiring them to take photos using their phone camera and upload the photo from their photo gallery. Participants rarely wrote the details of offline information in the description box provided.

#### 3.3.6. Recording their level of trust

When deciding on how much they trusted the information sources, some participants were unsure about how to answer. Some participants estimated their trust level relative to their own knowledge, the author of the information, and the source type. Some of the older participants had problems dragging the sliding scale of trust to a specific percentage and took several attempts to move it to the percentage they wanted. One participant preferred the trust scale initially be placed at 0% instead of 50% because they wanted to move the slider to around 50–70%.

## 4. Discussion

Our findings showed that the diary tool built as a component of the IDP is a usable and useful way to capture topic-specific information access and exposure from study participants, which could then be directly connected to outcome measures such as medication adherence. We found that participants often wanted to use the diary tool in ways that were not intended, so there is a need to maintain the core purpose of the tool for observing behaviors while also ensuring that participants want to continue to use the diary tool appropriately throughout a study observation period.

The diary tool can provide more detailed information about the health information that people access than is possible with traditional one-off administered questionnaires about media use (29). Traditional forms of media use diaries have been used in health research to collect both intentional searching and unintentional exposure to health information (30, 31). The diary tool made it easier for participants to record topic-specific information compared to traditional media use diaries by reducing the effort required. However, since the diary tool requires that participants consciously consider and record relevant information as they encounter it, it also has the potential to influence how participants access and appraise health information during a study observation period. For example, it can make them more aware of what they access, how much information they access, and how they appraise the information they encounter (29).

We discovered that participants using the information diary tool wanted to use it in ways that were not anticipated as part of the design of the diary tool for observational study designs. For example, users reported wanting to use it to discuss health information sources with their doctor, to access credible information, and as a guide to appraise health information. Marien et al. (32) reported a similar finding when tailoring digital interventions to meet the needs of users while also meeting the primary needs of researchers. As intrinsic motivation is a critical factor driving participation in research (33), participants users are likely to be more motivated to engage in research when they perceive the value of the study in addressing their health needs and interest (34). For observational studies, it is important to find a balance between making the diary tool useful for participants so they want to continue to use it throughout a study, while minimizing the potential impact it has on their behaviors.

In our study, study participants have become more aware of the need to appraise health information when they recorded their trust level in the diary tool. This phenomenon was supported by French and Sutton (35), who showed that study participants who are part of the activity of measurement can become more aware of their thinking and feelings. Our study suggested a potential usefulness of the diary tool to remind users to think again the credibility of the health information by just asking them to decide the trust level. Trust in health information depends on how a user perceives credibility and reliability (36), which is in turn influenced by health literacy and their affect toward the information (37). Since trust is an important factor that is likely to be related to how people make sense of information and use it to support their decision making (38), we thought that it was important to keep trust as a component of each record.

This study highlights that an appropriate design for information diaries requires a careful balance between providing features that users want and constraints that minimize the potential impact of the tools on behavior during a study (39). For example, additional features that support critical appraisal of information sources might be valuable for users but may limit the potential for the tool to be used to identify risk signals associated with potential harms such as non-adherence to medication. Our approach to the changes is to limit the number of “behavior-modifying” features that users have access to during a period of observation, and then unlock them to support appraisal and access to evidence-based health information after completion of the data collection.

From the usability evaluation, we learned that users had difficulty with how we described the main categories. It is important to be able to capture the difference between users actively seeking information (both online and offline) and inadvertently being exposed to health information while performing unrelated activities. To effectively capture the differences, future versions of the diary tool would choose the source of the information first (online sources such as a post on a Facebook page, or an advertisement on a webpage, or offline sources such as a consultation with a doctor or discussing with a family member), and then flag whether they were searching for health information or unintentionally encountered it while doing something else.

A key strength of the study was that participants were relevant to topic of study, with high cardiovascular risk and mostly from older age groups. A second strength of the study included the location of Malaysia, a multiracial country with multiple languages, which makes it a useful setting for evaluating the localization of the diary tool and how we made it available in multiple languages.

One limitation of the study was the possibility of selection bias, given that a high proportion of patients declined to participate in this study. Majority of those who participated in this study had at least a degree and, hence, they may have a higher digital literacy, more familiar with using a smartphone and be more inclined to seek high-quality sources of information. A second limitation was related to analyzing whether users were capturing all instances of topic-relevant information they encountered. We had no way of triangulating what they recorded against any form of ground truth observation.

## 5. Conclusion

The diary tool developed as part of the IDP serves its function as a way of capturing the topic-specific information study participants encounter. Our study showed that use of the diary tool might influence health information-seeking and information appraisal behavior. The diary tool met requirements for usability and can provide a more detailed view of the health information that people encounter compared to traditional methods such as questionnaires. Future development of information diary tools should consider the balance between providing features that users want and the requirements of observational studies that seek to capture information access and exposure.

## Data availability statement

The original contributions presented in the study are included in the article/Supplementary material, further inquiries can be directed to the corresponding author.

## Ethics statement

The studies involving human participants were reviewed and approved by University of Malaya Medical Center Medical Research Ethics Committee (MECID No: 2021324-9981). The patients/participants provided their written informed consent to participate in this study.

## Author contributions

HL, AD, CN, and AA contributed to the conception and design of the study. HL, AD, CN, AA, and JD contributed to developing research tools. HL, WL, and KL were involved in data collection and interviews. HL, WL, AD, and CN performed the data analysis. HL wrote the drafts of the manuscript. All authors contributed to reviewing and editing the submitted version. All authors contributed to the article and approved the submitted version.
