# Supplementary material for: Utility and usability evaluation of an information diary tool to measure health information access and exposure among patients with high cardiovascular risk
Source: Front Public Health. 2023 May 9;11:1132397. doi: 10.3389/fpubh.2023.1132397 (PMC10203480; doi:10.3389/fpubh.2023.1132397)
Supplement: Supplementary file 1 [file Table_1.DOCX]

# Appendix 1:

# INTERVIEW GUIDE

Preamble:

- Ice-breaking session (introduce and get to know participants)
- Explain the purpose of the session (to find out your views about IDP)
- Interested in personal views and opinions
- No right or wrong answers
- Permission to refuse answer questions
- Views will be kept confidential
- Obtain permission for audiotaping.
- Explain the confidentiality of recordings and data.
- Get demographic data and take written consent (for both interview and audiotaping)
- Any questions before we start?

| General question | How would you describe your overall experience after using IDP for a week? |
| --- | --- |
| **Nielsen’s usability domains** | |
| Learnability  (How easy is it for users to accomplish basic tasks the first time they encounter the design?) | How easy or difficult was it for you to record your encounters with statin information?  What features do you find most valuable and why? |
| Efficiency  (Once users have learned the design, how quickly can they perform tasks?) | Once you had learned how to use IDP, how easy was it for you to do it by yourself?  Can you give me a few examples of situations when you would use the tool? |
| Memorability  (When users return to the design after a period of not using it, how easily can they re-establish proficiency?) | When you did not use IDP for XX period (depends on patients’ data), how easy for you to enter the information into it? |
| Errors  (How many errors do users make, how severe are these errors, and how easily can they recover from the errors?) | Did you encounter any times where you were unable to record the information you saw in the tool?  If you could change one thing about the tool, what would it be and why? |
| Satisfaction  (How pleasant is it to use the design?) | What do you think about the design?  Are you satisfied with it? Why? |
| **Utility** | What do you think of the functionality of IDP as a patient?  Do you think IDP is useful or not useful? Why?  What will make you continue to use this IDP? |
| **Specific tasks in IDP:** Please tell me what you think about performing the tasks. | |
| To register in IDP with an email address. | |
| Entering information sources   - Online search - Online browsing - Offline | |
| Decide the level of trust using the dragging scale. | |
| Describe what you saw in free text | |
| Attach screenshot | |
