# Supplementary material for: Utility and usability evaluation of an information diary tool to measure health information access and exposure among patients with high cardiovascular risk
Source: Front Public Health. 2023 May 9;11:1132397. doi: 10.3389/fpubh.2023.1132397 (PMC10203480; doi:10.3389/fpubh.2023.1132397)
Supplement: Supplementary file 2 [file Table_2.DOCX]

**Appendix 2.** Total submissions of health information in the Information Diet Platform (IDP).

| **Participant** | **Categories** | | | **Total no. of submission** |
| --- | --- | --- | --- | --- |
|  | **Online search** | **Online Browsing** | **Offline** |  |
| P1 | Google (n=5) | YouTube (n=3) |  | 8 |
| P2 |  |  |  | 0 |
| P3 | Google (n=1) |  | Doctor (n=1)  Newspaper (n=6) | 8 |
| P4 | Google (n=4)  Yahoo (n=3)  Website (n=2) | WhatsApp (n=3)  The Star online (n=2) | Book (n=1) | 15 |
| P5 |  | WhatsApp (n=8) |  | 8 |
| P6 | Google (n=1) | WhatsApp (n=3)  Facebook (n=3) |  | 7 |
| P7 |  | Facebook (n=6) | Friends (n=1) | 7 |
| P8 | Google (n=1) | Facebook (n=2) | Newspaper (n=4) | 7 |
| P9 | Google (n=27)  Website (n=1) | Facebook (n=2) | Billboard (n=9) | 39 |
| P10 | Google (n=1) | WhatsApp (n=1)  YouTube (n=1)  TikTok (n=4) |  | 7 |
| P11 | Google (n=6) | WhatsApp (n=1)  YouTube (n=1)  Facebook (n=1) | Newspaper (n=1) | 10 |
| P12 | Google (n=1) | The Star online (n=1)  WhatsApp (n=1) | Newspaper (n=1)  Pamphlet (n=1) | 5 |
| P13 | Google (n=2) |  |  | 2 |
| P14 | Google (n=7) | WhatsApp (n=3) |  | 10 |
| P15 | Google (n=4) | Instagram (n=1) |  | 5 |
| P16 | Google (n=13) | Facebook (n=192)  YouTube (n=14)  Instagram (n=11) |  | 230 |
| P17 | Google (n=4) |  |  | 4 |
| P18 | Google (n=4) | Instagram (n=5)  WhatsApp (n=1)  YouTube (n=1) |  | 11 |
| P19 | Cari (n=2) | YouTube (n=1) | Newspaper (n=4)  Book (n=3)  Friends (n=1) | 11 |
| P20 | Website (n=3)  Google (n=1)  Baidu (n=1) | Facebook (n=12)  YouTube (n=2) | Newspaper (n=4) | 23 |
| P21 | Google (n=1) | Facebook (n=8)  WhatsApp (n=1) | Newspaper (n=1)  Book (n=1) | 12 |
| P22 | Google (n=9) | Facebook (n=3)  WhatsApp (n=1) |  | 13 |
| P23 | Website (n=1) | WhatsApp (n=6) |  | 7 |
| P24 | Website (n=1)  Google (n=1) | Twitter (n=1) |  | 3 |
